# Incomplete Kawasaki Disease in an Adult South Asian Patient

**DOI:** 10.1177/2324709618792028

**Published:** 2018-07-24

**Authors:** Neetu Boodoosingh, Rajeev Seecheran, Saleem Varachhia, Narine Mack, Vinay Minocha, Stanley Giddings, Naveen Anand Seecheran

**Affiliations:** 1 South West Regional Health Authority, San Fernando, Trinidad and Tobago; 2 University of the West Indies, St Augustine, Trinidad and Tobago

**Keywords:** incomplete Kawasaki disease, incomplete Kawasaki syndrome, South Asian, adult

## Abstract

Kawasaki disease is an acute multisystemic vasculitis occurring predominantly in children and rarely in adults, with sequelae of potentially life-threatening coronary artery aneurysms. “Incomplete” Kawasaki disease is a novel concept and considered a diagnosis of exclusion as it alludes to patients with fever lasting ⩾5 days and 2 or 3 clinical criteria without another reasonable explanation for the illness. The multidisciplinary team should be vigilant for this oligosymptomatic clinical presentation, specifically within this subgroup despite age and ethnicity, and the syndrome should be considered as a differential diagnosis in challenging cases presenting as infectious or autoimmune disease.

## Introduction

Kawasaki disease (KD) is an acute multisystemic vasculitis occurring predominantly in children and rarely in adults with sequelae of potentially life-threatening coronary artery aneurysms (CAAs).^1,2^ The precise etiology is yet to be ascertained; however, epidemiologic studies have implicated infectious agents with both autoimmune and genetic mechanisms being postulated as well.^3^ The pathophysiology involves a complex inflammatory milieu with a predilection for small- to medium-sized arteries, especially the coronary vessels.^4,5^

It is estimated that there are approximately 10 000 incident cases per year in Japan alone and 4000 in the United States.^6,7^ The epidemiology and characteristics of this enigmatic syndrome are virtually unknown in the largely heterogeneous Caribbean population; however, it remains the leading cause of acquired heart disease in the developed world. The most devastating complication is that of CAA, but also include other organ systems.^8^

The diagnosis is usually clinched via guidelines as there is no specific, confirmatory test available. “Incomplete” KD is a novel concept and considered a diagnosis of exclusion as it alludes to patients with fever lasting ⩾5 days and 2 or 3 clinical criteria without another reasonable explanation for the illness.^2^ The term “atypical” KD should be reserved for patients who display symptoms that are not common in classical KD, such as renal impairment, acute surgical abdomen, and pleural effusion.^8^

We describe a first case report of an adult South Asian patient with incomplete features of KD, which can masquerade as a clinical distractor.

## Case Report

**Figure 1.**
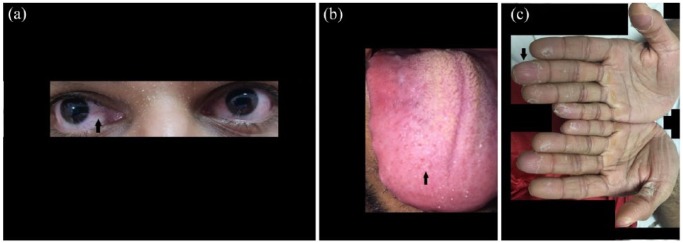
(a) The patient’s bilateral keratoconjunctivitis with chemosis, indicated by the black arrow. (b) The patient’s strawberry tongue glossitis with hyperplastic fungiform papillae, indicated by the black arrow. (c) The patient’s palmar desquamation with incomplete dehiscence of the epidermis and associated ichthyosis, indicated by the black arrow.

A 29-year-old South Asian male with no significant medical history presented to the emergency department with a 14-day symptom complex of persistent, high-grade fever refractory to antibiotics and antipyretics, malaise, and anorexia with a 10-pound weight loss. There were no recent medications, ill contacts, or travel history. His vital signs affirmed normotensive blood pressures, a resting sinus tachycardia of 110 beats per minute, and pulse oximetry of 98% on room air with a mild pyrexia of 38.8°C. Physical examination revealed bilateral conjunctivitis with chemosis, a strawberry tongue glossitis, palmar desquamation, and ichthyosis (see Figure 1a-c, respectively). There was no evidence of lymphadenopathy or dermatologic manifestations, such as rash.

Recent pertinent laboratory investigations (see Table 1) included a leukocytosis and notable thrombocytosis, normal comprehensive metabolic panel, markedly elevated inflammatory markers of erythrocyte sedimentation rate, and C-reactive protein. An extensive infectious disease diagnostic workup indicated negative blood, urine, and stool cultures and normal tests for human immunodeficiency virus, mycobacterium tuberculosis, hepatitis B and C, influenza A and B, adenovirus, echovirus, coxsackie virus, dengue, malaria, leptospirosis, mycoplasma, legionella, Epstein-Barr virus, cytomegalovirus, and *Clostridium difficile* toxin. An in-depth immunological panel revealed no evidence of vasculitides or rheumatological disease, such as systemic lupus erythematosus, rheumatoid arthritis, Sjogren’s syndrome, polyarteritis nodosa, the polyangiitis spectrum, and cryoglobulinemia. A potential adverse drug reaction was not entertained as the patient was not administered any recent therapeutic or complementary alternative agents. Cardiovascular testing with both an electrocardiogram and echocardiogram were normal and advanced imaging with a pan-body computed tomography scan was also unremarkable. He was deemed to have an incomplete presentation of KD and was initiated on high-dose enteric-coated aspirin (Bayer HealthCare Pharmaceuticals LLC, Berlin, Germany) 325 mg every 8 hours, as well as single infusion of intravenous immunoglobulin (GammaGard, Baxter International Inc, Glenview, IL) at a dose of 2 g/kg over a 12-hour period. Subsequently, his clinical syndrome gradually resolved over the ensuring hospitalization as his pyrexia de-effervesced along with steady improvement of his inflammatory markers. He did not receive any glucocorticoids or immunomodulating therapies. He was safely discharged after 1 week of inpatient care on low-dose aspirin monotherapy with gastroprotective proton-pump inhibitors and subsequently scheduled for a dedicated cardiac computed tomography angiogram that did not reveal any CAAs at a later outpatient clinic appointment (2 weeks from index hospitalization).

**Table 1.** Comprehensive Laboratory Testing Including the Infectious and Immunologic Panels.

Tests Performed	Result	Reference Range
Complete blood count, comprehensive metabolic panel		
White cell count	16.1 × 109/L	4.5-11.0 × 109/L
Hemoglobin	12.9 g/dL	14.0-17.5 g/dL
Platelet count	606 × 103/µL	156-373 × 103/µL
Serum potassium	4.1 µmol/L	3.5-5.1 µmol/L
Serum sodium	136 µmol/L	135-145 µmol/L
Serum creatinine	0.7 mg/dL	0.5-1.2 mg/dL
Blood urea nitrogen	10 mg/dL	3-20 mg/dL
Fasting blood sugar	80 mg/dL	60-120 mg/dL
Alanine aminotransferase	90 IU/L	20-60 IU/L
Aspartate aminotransferase	35 IU/L	5-40 IU/L
Total bilirubin	0.9 mg/dL	0.2-1.2 mg/dL
Alkaline phosphatase	120 U/L	40-129 IU/L
Albumin	3.4 g/dL	3.5-5.5 g/dL
Albumin-corrected calcium	9.6 mg/dL	9.6-11.2 mg/dL
Infectious diseases panel		
Erythrocyte sedimentation rate	60 mm/h	0-22 mm/h
C-reactive protein	90 mg/dL	0.0-1.0 mg/dL
Blood cultures	Negative	Positive or negative
Urine culture	Negative	Positive or negative
Stool culture	Negative	Positive or negative
Stool ova, cyst, and parasites	Negative	Positive or negative
Human immunodeficiency virus enzyme-linked immunosorbent assay	Nonreactive	Nonreactive or reactive
Venereal disease research laboratory test	Nonreactive	Nonreactive or reactive
QuantiFERON-TB GOLD (Cellestis Limited, Carnegie, Victoria, Australia)	Negative	Positive or negative
Hepatitis B surface antigen	Negative	Positive or negative
Hepatitis C immunoglobulin M (IgM) antibodies	Negative	Positive or negative
Hepatitis C Immunoglobulin G (IgG) antibodies	Negative	Positive or negative
Influenza A and B nasal swabs	Negative	Positive or negative
Adenoplus (Quidel Corporation, San Diego, CA)	Negative	Positive or negative
Echovirus antibodies (6,7,9,11, and 30)	<1:10	<1:10
Coxsackie B virus antibodies (B1-B6)	<1:10	<1:10
Dengue IgM antibodies	Negative	Positive or negative
Dengue IgG antibodies	Negative	Positive or negative
Malaria thick and thin smears	Negative	Positive or negative
Leptospirosis IgM antibodies	Negative	Positive or negative
Mycoplasma IgM antibodies	Negative	Positive or negative
Mycoplasma IgG antibodies	Negative	Positive or negative
Urine Legionella antigen	Negative	Positive or negative
Heterophile antibody test	Negative	Positive or negative
Epstein-Barr virus IgM antibodies	Negative	Positive or negative
Epstein–Barr virus IgG antibodies	Negative	Positive or negative
Cytomegalovirus IgM antibodies	Negative	Positive or negative
Cytomegalovirus IgG antibodies	Negative	Positive or negative
Stool clostridium difficile toxin A/B	Negative	Positive or negative
Antistreptolysin O Titer	90 IU/mL	0-200 IU/mL
Immunologic and rheumatologic panel		
Antinuclear factor	Negative	Positive or negative
Anti–double stranded deoxyribonucleic acid antibodies	<30.0 U/mL	<30.0 U/mL (negative)
C3	190 mg/dL	83-193 mg/dL
C4	43 mg/dL	15-75 mg/dL
Anti–cyclic citrullinated peptide antibodies	<20.0 U/mL	<20.0 U/mL (negative)
Rheumatoid factor	Negative	Positive or negative
Extractable nuclear antigen panel including anti-RNP, -Ro, -La, -SCL-70, -Jo1, and –centromere	All negative	Positive or negative
Perinuclear anti-neutrophil cytoplasmic antibodies	5.42 U/mL	<10.0 U/mL (negative)
Cytoplasmic anti-neutrophil cytoplasmic antibodies	3.73 U/mL	< 10.0 U/mL (negative)
Cryoglobulin blood test	Negative	Positive or negative

## Discussion

The diagnosis of classic KD is usually verified via guidelines as there is no specific, confirmatory test available (Table 2).^8-10^ Adult incomplete KD was clinically diagnosed based on the absence of overt infection, persistence, and recrudescence of high-grade fever despite empirical antibiotics and antipyretics, and the presence of conjunctivitis, glossitis, and palmar desquamation.^9,11^ A recent French study evaluated 9 patients who fulfilled criteria for incomplete disease. The median time to diagnosis was 13 days, which coincided with our patient’s time to presentation and the main symptoms were fever (100%), exanthema (98%), changes in the extremities (91%), conjunctivitis (77%), oral cavity changes (89%), cervical adenitis (55%), and cardiac abnormalities (45%) of which fever, changes in the extremities, conjunctivitis, and oral cavity changes featured as clinical signs in our patient. Overall, 35% of the patients showed large-vessel vasculitis: coronary vasculitis (26%) and coronary aneurysm (19%), neither of which were replicated in our patient.^11,12^ Another large, international-based registry of non–East Asian incomplete KD patients reported statistically significant increases in the occurrence of conjunctivitis, mucosal changes, and extremity alteration, which paralleled our observations.^12^

**Table 2.** Criteria for Diagnosis of Kawasaki Disease.^8-10^.

Fever ⩾5 days and ⩾4 days of the following:
• Rash: diffuse maculopapular eruption, diffuse erythroderma, or erythema multiforme-like rash
• Conjunctivitis: bilateral bulbar conjunctival injection without exudate
• Cervical lymphadenopathy: usually unilateral, ⩾1.5 cm lymph node, anterior cervical triangle
• Extremity changes: erythema and edema of the hands and feet in acute phase, desquamation of the fingers and toes usually begin in the periungual region in subacute phase
• Oral changes: erythema and cracking of lips, strawberry tongue with erythema, and prominent fungiform papillae, diffuse erythema of the oropharyngeal mucosa

To our knowledge, this is the first reported case of an adult South Asian male in the Caribbean presenting with incomplete KD. Apart from his age, our patient was also of South Asian ethnicity; and currently, there exists a paucity of literature with regard to this subgroup. In many developing countries, including India, the majority of patients with KD continue to remain undiagnosed likely attributed to lack of awareness among clinicians.^13^ Adult-onset KD should be considered as a differential diagnosis in challenging cases presenting as infectious or autoimmune disease even if the patient is not of East Asian lineage.^13-15^

The key therapeutic strategy for KD is to prevent the formation of CAAs and symptom alleviation. Inpatient supportive management and administration of intravenous immunoglobulin (IVIG) is considered to be the mainstay of treatment.^16-18^ Currently, there are several risk scores for IVIG resistance that could identify patients at high-risk for nonresponse to IVIG treatment, which in turn is highly associated with the development of CAAs.^19-21^

American Heart Association (AHA) guidelines recommend a second dose of IVIG, methylprednisolone, a longer tapering course of prednisolone or prednisone plus IVIG, cyclosporine, immunomodulatory monoclonal antibody therapy, cytotoxic agents, or plasma exchange for patients resistant to IVIG.^10,18,22^

Aspirin has been the conventional, standard therapy for its antiplatelet effects, initially a high-dose regimen for a variable period, followed by a lower dose for a protracted period in patients with small CAAs, whereas dipyridamole is indicated in patients with larger CAAs.^23^ It is recommended by the AHA guidelines that these patients should be treated with low-dose aspirin until aneurysms are documented to have regressed. Clopidogrel has also been used in cases of aspirin hypersensitivity.^4^

As of 2017, the AHA and the Japanese Circulation Society guidelines specify that KD patients require vigilant follow-up with noninvasive imaging and cardiac stress testing and to detect progressive stenosis, thrombosis, and luminal occlusion that may lead to myocardial ischemia and infarction.^10,24,25^

The literature is not replete with describing this subpopulation of incomplete KD with regard to age and ethnicity and this case emphasizes its rarity, but also underscores the absolute necessity for specific guidelines in this patient panel.^2^

## Conclusion

In summary, we describe the first case report of incomplete KD in an adult South Asian patient based in the Caribbean. The multidisciplinary team should be vigilant for this oligosymptomatic clinical presentation, specifically within this subpopulation despite age and ethnicity, and the syndrome should be considered as a differential diagnosis in challenging cases presenting as infectious or autoimmune disease.
